# A comparative study of one-stage posterior unilateral limited laminectomy vs. bilateral laminectomy debridement and bone grafting fusion combined with internal fixation for the treatment of aged patients with single-segment spinal tuberculosis

**DOI:** 10.1186/s12891-022-05562-9

**Published:** 2022-06-28

**Authors:** Liyuan Jiang, Xiaolong Sheng, Zhansheng Deng, Qile Gao, Shaohua Liu

**Affiliations:** 1 Department of Spine Surgery and Orthopaedics, Xiangya Hospital, Central South University, Changsha, 410008 China; 2 Key Laboratory of Organ Injury, Aging and Regenerative Medicine of Hunan Province, Changsha, 410008 China; 3 Clinical Research Center for Geriatric Disorders, Xiangya Hospital, Central South University, Changsha, China

**Keywords:** Spinal tuberculosis, Thoracic and lumbar tuberculosis, Unilateral limited laminectomy, Bilateral laminectomy, Aged

## Abstract

**Study design:**

This is a retrospective study.

**Background:**

To assess and compare the clinical outcomes of posterior unilateral limited laminectomy (ULL) or bilateral laminectomy (BL) debridement and bone grafting fusion combined with internal fixation among aged patients with single-segment thoracic and lumbar tuberculosis (SST/LTB).

**Materials and methods:**

We performed a retrospective study on aged patients (age > 65 years old) with SST/LTB from January 2010 to October 2018. We reviewed 36 aged patients who were treated with BL and 31 aged patients treated with ULL. All participants had undergone and finished a three-year follow-up. The outcomes were evaluated by the improvement of neurological function, correction Cobb angle, bone fusion time, and back pain, as well as operative time, blood loss, hospital stay, and postoperative complications.

**Results:**

The operative time, blood loss volume, and incidence of complications in group B were significantly less than those in group A (*P* < 0.01). The postoperative kyphotic angle in both groups was reduced significantly compared to the preoperative status (*P* < 0.01). The percentage of neurological improvement was 92.9% in group A and 90.9% in group B. All patients achieved solid bone fusion after surgery. At three-year follow-up, the angle loss in group B was significantly less than that in group A (*P* < 0.01); Furthermore, patients in group B had a lower average visual analog scale score of back pain and Oswestry Disability Index score than patients in group A (*P* < 0.05).

**Conclusions:**

For aged patients with SST/LTB, ULL is a safer and more effective surgical treatment than BL.

## Introduction

During the last decade, the incidence of spinal tuberculosis (TB) has been on the rise due to the aging of the population, the larger numbers of immunocompromised hosts, and the use of intravenous drugs [1–3]. The thoracic and lumbar levels are most frequently affected among cases of spinal TB [4, 5]. Most patients with spinal TB can be cured with conservative methods. However, for patients with developing spine instability and neurological impairment, medications combined with surgery are needed. Regretfully, there is no consensus of a mainstream surgery type for elderly individuals with thoracic and lumbar TB (T/LTB) [6–9].

For aged patients with TB, mortality is significantly high and is three times higher than that of younger adults during therapy [10]. The clinical presentation in aged patients with spinal TB has unique characteristics that are different from those in young patients. First, aged patients generally have poor conditions, such as malnutrition, anemia, and hypoalbuminemia [6]. Second, older adults have high percentages of drug resistance [3, 10]. Third, a high percentage of aged patients have multiple major comorbidities. Fourth, aged patients are prone to develop abscesses, caseous tissues, and sequestra, which can lead to spinal cord compression and nervous system damage. Last, there are a few cases of migrating abscesses in older patients. Hence, for aged patients who need surgical treatment, minimally invasive surgery is recommended for this vulnerable population.

Surgery for spinal TB aims to decompress the spinal cord, correct deformity, and restore the stability of the spine. The anterior approach is the gold-standard approach for debridement and decompression in Pott's spine, and it was popularized by Hodgson in 1960 [11, 12]. However, this operation method requires a long operation time and causes massive surgical trauma, which may increase the risk of serious surgical complications and extend patients' recovery time [13–15]. Elderly individuals with weak cardiovascular and respiratory systems are probably unable to withstand the trauma and complications caused by this operation method. With the development of posterior pedicle screw fixation, the posterior-only approach causing less surgical trauma has been widely accepted by an increasing number of spine surgeons [15–17]. It has been suggested to be a safe and effective approach for T/LTB, especially single-segment T/LTB (SST/LTB). However, bilateral laminectomy (BL) is always performed with the traditional posterior-only approach, which can destroy large-scale posterior columns, resulting in surgical spinal instability, epidural scar adhesions, and even post-laminectomy syndrome [18–20]. Many surgeons are still concerned that the large-scale destruction of the posterior column may lead to complications, decreasing surgical efficacy. The posterior unilateral limited laminectomy (ULL) is a minimally invasive technique which was used to treat different kinds of spinal diseases, such as spinal canal stenosis and septic thoracolumbosacral spondylodiscitis [21, 22]. However, very few articles reported its application to spinal TB treatment. To minimize the damage to the posterior column, we further applied a ULL to treat aged patients with SST/LTB. In the present study, we compared the clinical and radiological outcomes of ULL and BL for the treatment of aged patients with SST/LTB.

## Materials and methods

### Clinical information

To compare ULL with BL for the treatment of aged patients with SST/LTB, we performed a retrospective study in aged patients (aged 65 and older) with SST/LTB, and this study was approved by the Ethics Committee of Xiangya Hospital, Central South University, China. In this retrospective study, the medical records of inpatients admitted for SST/LTB to Xiangya Hospital, Central South University, from January 2010 to October 2018 were continuously reviewed. The diagnosis of spinal TB was based on clinical symptoms (back pain, night sweats, afternoon hot flashes, weight loss, and neurological dysfunction), laboratory tests (elevation of erythrocyte sedimentation rate (ESR), C-reaction protein (CRP)), radiological results (X-ray films, computed tomography, and magnetic resonance imaging), pathological findings, and positive bacterial culture.

Aged patients with SST/LTB who accepted either ULL or BL in the same surgical group as the surgical treatment in our medical system were consecutively selected with the following indications: the age of the patient was over 65 years old; spinal instability caused by bone destruction; progressive worsening of kyphosis; spinal cord compression by abscess or necrosis tissue; lesion confined to one functional spinal unit without rigid kyphosis and extensive anterior TB abscess.

### Preoperative preparation

Before both operation methods were performed, all patients routinely received the HREZ (isoniazid: 300 mg/day, rifampicin: 450 mg/day, pyrazinamide: 750 mg, and ethambutol: 750 mg/day) chemotherapy regimen for 2–4 weeks. For patients with severe malnutrition, anemia, and hypoproteinemia, nutritional treatment was given until the patients' anemia and hypoproteinemia were improved to normal levels. Surgery was performed after TB poisoning symptoms had been significantly relieved.

### Surgical procedure

#### Surgery for group A

**Fig. 1:**
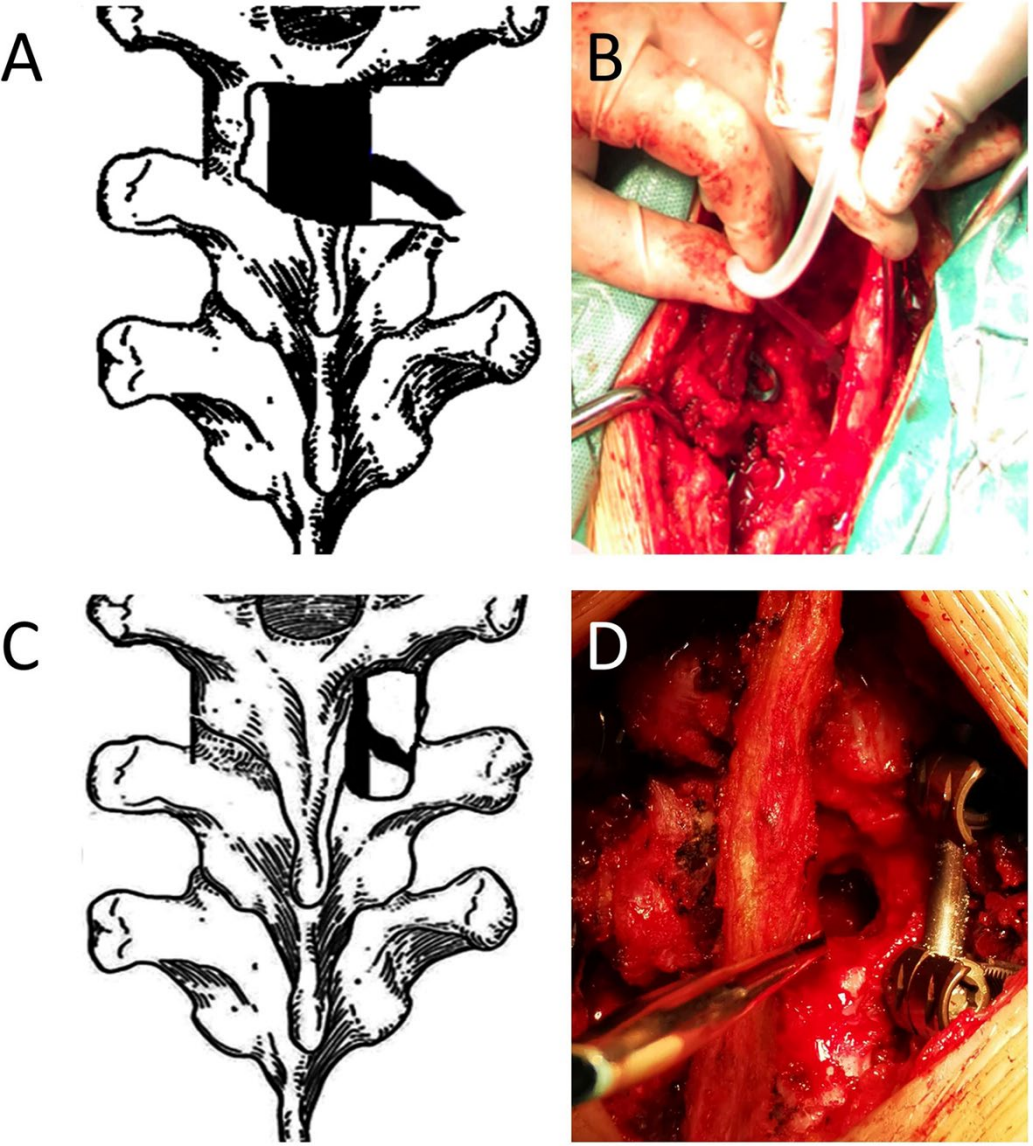
Scope of the excision in group A (**A**-B) and group B(**C**-**D**)

The patient was placed intravenous drugs in a prone position after general anesthesia with tracheal intubation. Through a midline incision, the involved posterior spinal elements, including the vertebral lamina and facet joints, were exposed. Transpedicular screws were allowed to be fixed on both sides of the vertebral lamina based on preoperative symptoms and imaging. After transpedicular screws were implanted, C-arm X-ray was used to confirm their accuracy. A temporary pre-bent rod was installed on the side with fewer lesions for temporary fixation during decompression and debridement. Then, we selected the severe side with a paraspinal abscess as the decompression and debridement side. The facet joints, vertebral lamina, spinous process, and a small part of the adjacent rib were removed (Fig. 1A-B). Then, the cartilage of the necrotic disc from the endplate of the collapsed vertebrae was removed until the healthy bleeding bone was observed using a curette. The decompression range was based on the extent of spinal canal stenosis and the scope of the paraspinal abscess. We accessed the paraspinal abscesses via the paravertebral sinus and carefully separated the abscess wall, removed pus, and scraped the necrotic tissue. After that, the deformity was slowly and carefully rectified with the help of compression and stretching of the internal fixation instrument. Allograft bone or autogenous bone was shaped for a posterior bone graft. Finally, 1.0 g of streptomycin and 0.2 g of isoniazid were locally administered, negative pressure drainage was placed in a suitable location, and incision sutures were performed.

#### Surgery for group B

We exposed the vertebral plate and performed transpedicular screw fixation as in group A. We performed ULL on the side with severe clinical manifestations. Focusing on the lesion, 1/3–2/3 of the inferior part of the upper lamina and 1/3–2/3 of the superior part of the lower lamina were resected to decompress the spinal cord and debride the lesion **(**Fig. 1C-D**)**. To expand the operative visual field, the operating table had been tilted 30° towards the non-decompression side. After a limited laminectomy, we removed pus and necrotic tissue and decompressed the spinal cord. Then, we opened the destructive intervertebral space and gradually removed the abscess, necrotic tissue, sequestrum, and sclerotic bone. Deformity rectification and the local application of anti-tuberculosis drugs were performed as in group A. The shaped allograft bone or autogenous bone was grafted in the intervertebral space. The defect area of the vertebral plate was covered with an allogeneic bone plate. Finally, negative pressure drainage placement and incision sutures were performed.

### Postoperative management

The drainage tube was removed when the drainage volume was less than 20 ml per 24 h. Patients in both groups continued the oral HREZ regimen postoperatively. Six months later, pyrazinamide was discontinued. Then, patients received nine- to 12-month regimens of HRE chemotherapy (6HREZ/9-12HRE).

### Measures of effectiveness and safety

Blood loss and operative time were recorded during the operation. The hospital stay and bone fusion time were recorded postoperatively. Preoperative, postoperative, and follow-up indicators, including the Cobb angle, neurologic status by American Spinal Cord Injury Association (ASIA) classification, ESR, CRP level, Oswestry Disability Index (ODI), and visual analog scale (VAS) for back pain, were measured and recorded at baseline and follow-up. Graft bone fusion status was evaluated according to the modified criteria of Lee et al. [21]. In addition, the following indicators were calculated: correction Cobb angle, angle loss, angle loss rate, and improvement rate neurological function. A three-year follow-up was required after our operation, and follow-up measurements were obtained during patients' return visits to the outpatient department. (Thoracic and thoracolumbar spine: correction Cobb angle = preoperative Cobb angle -postoperative Cobb angle; angle loss = last follow-up Cobb angle- postoperative Cobb angle; angle loss rate = angle loss/correction Cobb angle. Lumbar spine: correction Cobb angle = postoperative Cobb angle—preoperative Cobb angle; angle loss = postoperative Cobb angle—last follow-up Cobb angle; angle rate rate = Angle loss/correction Cobb angle. Improvement rate = number of patients with an improvement in ASIA classification/total number of patients with spinal cord dysfunction).

### Statistical analyses

The results were analyzed using SPSS software version 19.0 (SPSS Inc., Chicago, IL). T-tests were used to compare the changes in the indices within each group preoperatively, postoperatively, and at the follow-up. The Wilcoxon signed-rank test was used to analyze neurologic function preoperatively and at the final follow-up. The clinical data between the two groups were compared using Student's t-test. A discrepancy in normal data distributions was analyzed using a rank-sum test. When *P* < 0.05, the difference was statistically significant.

## Results

### Demographics and clinical characteristics

**Fig. 2:**
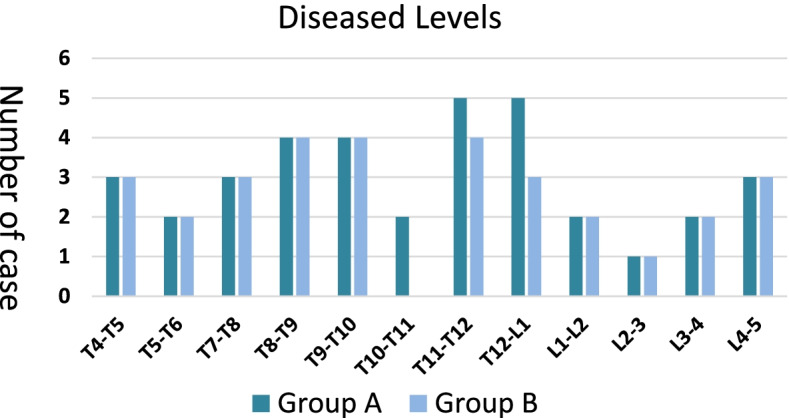
Diseased segment distribution

Within the study time frame, 73 qualified aged patients were selected. Complete follow-up data were available in 67 patients (91.8%), and 6 patients (8.2%) were lost during follow-up. We finally analyzed 67 inpatients with SST/LTB. Among these, 36 patients were treated with BL, and 31 patients were treated with ULL. Table 1 shows the demographics and clinical characteristics of the studied patients. The involved spine segments are presented in Fig. 2. The number involved T4/T5, T5/T6, T7/T8, T8/T9, T9/T10, L1/L2, L2/L3, L3/L4, L4/L5 respectively were three, two, three, four, four, two, one, two, three in both groups. There were no significant differences in sex, age, or spinal cord dysfunction between the two groups preoperatively (*P* > 0.05).

**Table 1:** Clinical data of patients

	group A	group B	*P-value*
Gender(Male/Female)	20/16	17/14	0.26
Average age(year)	71.3 ± 4.5	69.9 ± 3.6	0.19
Spinal cord dysfunction	77.8%	71.0%	0.81
Operation time(min)	192.2 ± 18.5	160.3 ± 28.1	< 0.01
Blood loss(ml)	611.9 ± 58.9	469.3 ± 85.3	< 0.01
Postop hospital stay(day)	14.5 ± 2.2	12.1 ± 2.4	< 0.01
Duration of follow-up(months)	43.33 ± 8.6	39.6 ± 10.1	0.10

Gender distribution was analyzed by Chi-square test; Age, operation time, blood loss, post-op hospital stay, and duration of follow-up were analyzed by Student's t-test. Spinal cord dysfunction was analyzed by Wilcoxon signed-rank test

*Postop* Postoperative

### Surgical condition (Table 1)

The mean operation time (192.2 ± 18.5 min), postoperative hospital stay (14.5 ± 2.2 days), and blood loss volume (611.9 ± 58.9 ml) in group A were significantly higher than the corresponding indicators (160.3 ± 28.1, 12.1 ± 2.4, 469.3 ± 85.3) in group B (all *P* < 0.01).

### Complications (Table 2)

**Table 2:** Complications

Complications	Group A	Group B
Superficial wound infections	3	1
Deep wound infections	1	1
DVT of lower limb	2	0
Cerebrospinal fluid leakage	2	0
Mild intestinal obstruction	4	2
Mild pressure sores	3	1
Total	15(41.7%)	5(16.1%)

*DVT* Deep Vein Thrombosis. The comparison of complications between groups A and B was performed by chi-square test (*P* = 0.03)

The details of the complications are shown in Table 2. In group A, complications occurred in 41.7% (15 out of 36) of the patients, which was significantly higher than the complication rate of 16.1% (5 out of 31) in group B (*P* = 0.03).

### VAS score for back pain and ODI score (Table 3)

**Table 3:** VAS score for back pain and Oswestry Disability Index score in two groups

Group	Visual Analog Scale score		Oswestry Disability Index	
	pre-OP	LF	pre-OP	LF
A	6.5 ± 1.0	1.8 ± 0.7	38.9 ± 6.2	24.8 ± 8.0
B	6.7 ± 1.1	1.2 ± 0.6	37.8 ± 5.9	20.6 ± 7.7
	*P* = *0.47*	*P* < *0.01*	*P* = *0.45*	*P* = *0.036*

Preoperative and postoperative comparison of VAS score and ODI between group A and group B was used the Student's t-test

*Preop* Preoperative, *LF* Last follow-up point

The details of the VAS score for back pain and ODI scores are shown in Table 3. Compared with the preoperative values, the mean VAS and ODI scores in both groups decreased at the final follow-up (*P* < 0.01). At the final follow-up, the mean VAS and ODI scores in group B were lower than those in group A (*P* < 0.05).

### Radiological data (Table 4)

**Table 4:** Cobb angle data in two groups

Groups	Kyphosis angle (°)			Correction Cobb angle(°)	Angle loss	Bone fusion time(mons)
	Preop	Postop	LF		Loss(°)	
A	26.1 ± 9.1	12.1 ± 3.1	13.7 ± 2.5	16.7 ± 5.5	2.6 ± 1.2	8.2 ± 1.9
B	25.5 ± 8.4	11.7 ± 4.2	13.1 ± 3.3	15.7 ± 6.9	1.9 ± 0.9	7.8 ± 1.5
	*P* = *0.77*	*P* = *0.73*	*P* = *0.45*	*P* = *0.81*	*P(0.01)* < *0.05*	*P* = *0.44*

*Postop* Postoperative, *Preop* Pre-operative, *LF* Last follow-up

Thoracic and thoracolumbar spine: Correction Cobb angle = preoperative Cobb angle -postoperative Cobb angle; Angle loss = last follow-up Cobb angle- postoperative Cobb angle; Angle rate rate = angle loss/ correction Cobb angle

Lumbar spine: Correction Cobb angle = postoperative Cobb angle—preoperative Cobb angle; Angle loss = postoperative Cobb angle—last follow-up Cobb angle; Angle rate rate = angle loss/ correction Cobb angle

**Fig. 3:**
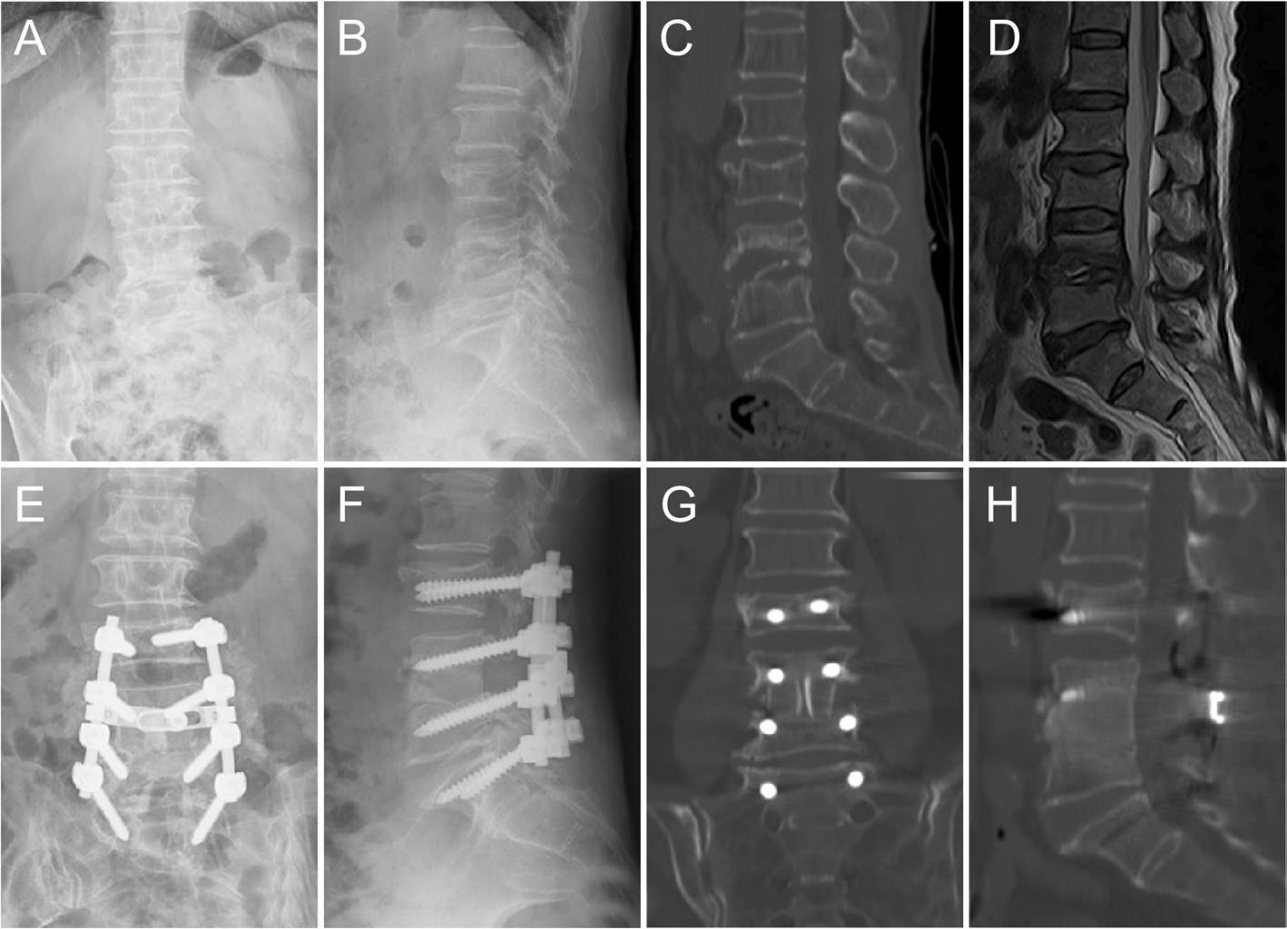
An old male with destructive L4-L5 was treated by posterior bilateral laminectomy debridement and bone grafting fusion combined with internal fixation. Figures 3**A**, **B**, **C**, and **D** are preoperative radiological images showing destructive bone at the L4 -5. Two years after the operation, the X-ray images (**E**, **F**) and CT images (**G**, **H**) show solid bone fusion

**Fig. 4:**
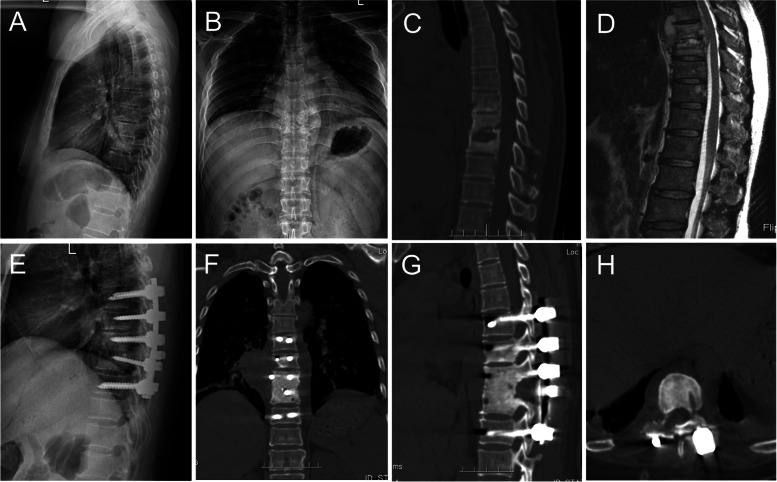
An aged male with destructive T8-9 was treated by posterior unilateral limited laminectomy debridement and bone grafting fusion combined with internal fixation. Figure 4 **A**, **B**, **C**, and **D** are preoperative images showing spinal canal abscess and destructive vertebra. Two years after the operation, the X-ray (**E**, **F**) and CT (**G**, **H**) showing correction of the Cobb angle and solid fusion of vertebral body and lamina

**Fig. 5:**
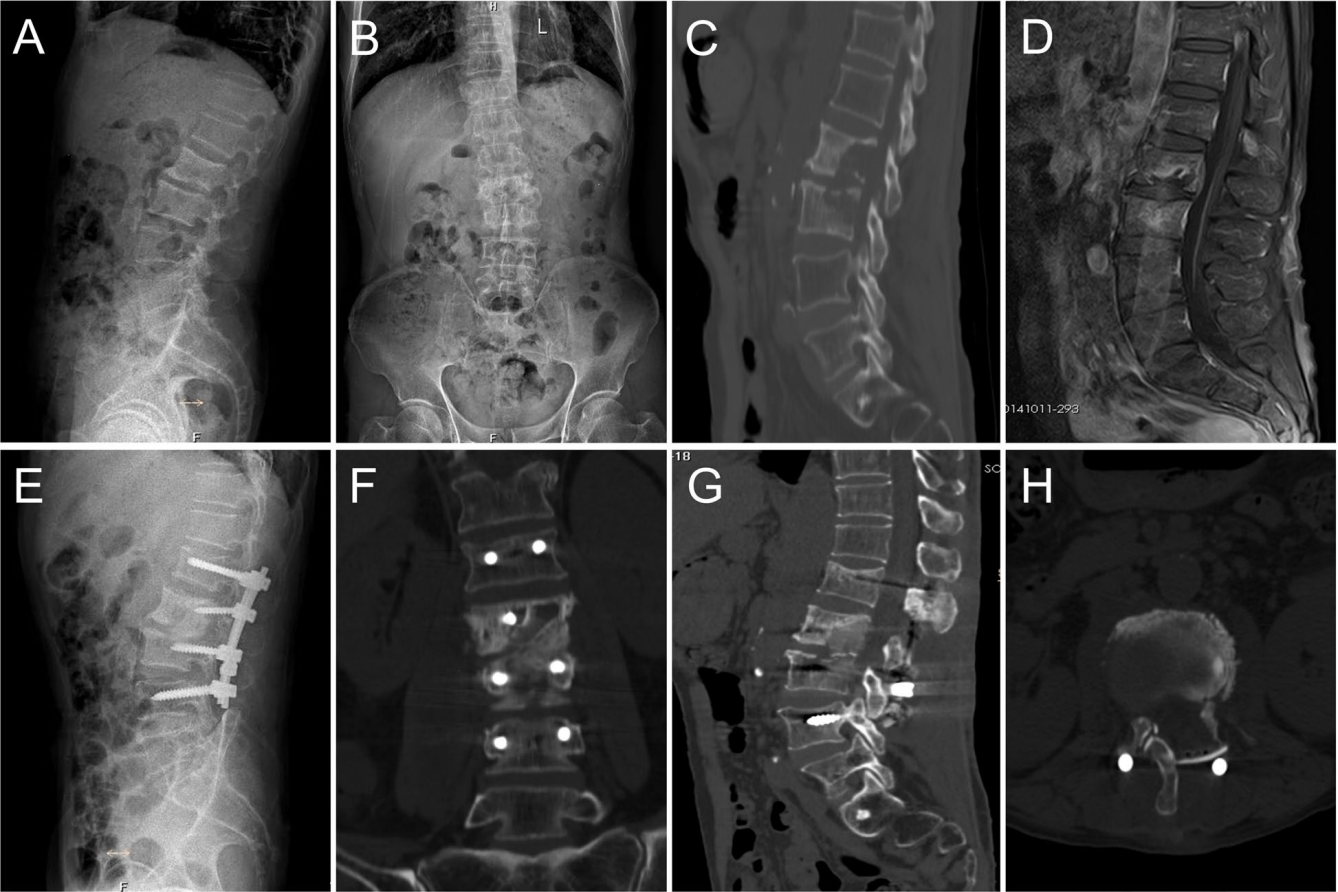
An aged male with destructive L2-3 was treated by posterior unilateral limited laminectomy debridement and bone grafting fusion combined with internal fixation. Figure 5 **A**, **B**, **C**, and **D** are preoperative images showing lesion and destructive vertebra at the L2 and L3 vertebrae. Two years after the operation, the X-ray (**E**) and CT (**F**, **G**, **H**) showed a solid bone fusion

Table 4 shows the mean Cobb angle at various time points in groups A and B. The mean Cobb angle did not significantly differ between groups A and B preoperatively and postoperatively (*P* > 0.05). There was no significant difference in the correction Cobb angle between the two groups (*P* > 0.05). At the last follow-up, group B had a smaller mean angle loss (1.9 ± 0.9°) than group A (*P* < 0.05). X-ray and CT showed that bony fusion was achieved in all patients. The mean bone fusion time was 8.2 ± 1.9 months in group A (Fig. 3) and 7.8 ± 1.5 months in group B (Figs. 4 and 5). There was no significant difference in bone fusion time between the two groups (*P* > 0.05).

### Laboratory data

The preoperative ESR and CRP values were 50.1 ± 21.7 mm/h and 43.2 ± 18.1 mg/l in group A and 46.4 ± 15.4 mm/h and 47.0 ± 16.6 mg/l in group B (*P* > 0.05). The ESR and CRP values in all patients returned to normal levels six months after surgery.

### Neurologic function (Table 5)

**Table 5:** Preoperative and postoperative ASIA classification in two groups

ASIA classification	Group A			Group B		
	Preop	LF	Improvement	Preop	LF	Improvement
A	0	0	0	0	0	0
B	4	1	3	3	1	2
C	6	4	5	7	3	6
D	18	3	18	12	3	12
E	8	28		9	24	

According to ASIA classification, spinal cord function improvement rate: Group A was 92.9%, and Group B was 90.9%. The *p*-value of comparison between groups A and B was calculated by Wilcoxon signed-rank test. There was no significant difference between the two groups (preoperation: *P* = 0.86; last follow-up point: *P* = 0.98)

*Postop* Postoperative, *LF* Last follow-up

The neurologic function in the two groups was evaluated via the ASIA grading system. There was no significant difference between the two groups in the ASIA grade before or after the operation (*P* > 0.05). There were 26 patients and 20 patients who demonstrated neurological improvement in group A and group B, respectively. The percentage of spinal cord function improvement was 92.9% in group A and 90.9% in group B at the last follow-up (*P* > *0.05*).

## Discussion

In the present study, our data showed that both BL and ULL were effective surgical treatments for aged patients with SST/LTB. These two approaches achieved similar results in the correction of the Cobb angle and neurological function improvement. However, after systematically comparing the two groups, we found that ULL achieved better clinical outcomes and was proven to be a more minimally invasive surgical technique with safer results.

First, ULL led to significantly lower operative time, less blood loss, a lower rate of complications, and a shorter duration of hospital stay than BL. In previous studies, Thome C et al. and Postacchini F et al. also compared ULL with BL for the treatment of lumbar spinal stenosis. Although they were applied in other spinal diseases, our study reinforced their conclusion that ULL caused less surgical trauma and achieved a faster postoperative recovery than BL [23, 24]. Second, the angle loss in patients who underwent ULL was smaller than that in patients who underwent BL. According to the three-column theory, the posterior spinal column plays a vital role in maintaining spinal stability and resisting shear, rotational, and compressive forces [25, 26]. Bresnahan L et al. pointed out that the removal of midline structures (i.e., spinous processes, vertebral arches, and interspinous and supraspinous ligaments) may contribute to the instability of the spine [27]. Biomechanical experiments have also proven that laminar reconstruction can maintain the integrity of the posterior column and effectively share the axial load of the instrumentation [28]. All these points of view indicate that preserving the posterior column is essential for maintaining the stability of the spine. Moreover, due to the reduced bone density, the stability of pedicle screw fixation in aged patients decreases to different levels [29, 30]. Therefore, maximum retention of posterior elements and laminar reconstruction was necessary for old patients with SST/LTB. Third, patients who underwent ULL had a lower back pain VAS score and ODI score than patients who received BL. BL always results in laminectomy membrane formation, which is recognized as a complication of spinal surgery that can cause a lack of recovery or even the worsening of neurologic conditions after surgery [31, 32]. After BL, fibrous scar tissue fills the bone defect area, forming a dense scar tissue membrane. In this membrane, fibrous connective tissue abnormally proliferates and adheres to the dura and nerve roots [33, 34]. Such adhesion around the nerve roots may cause considerable postoperative disorders [35], and the pain degree may be correlated with the amount of scar tissue [36]. In addition, such adhesions can also lead to new spinal cord compression, which can lead to substantial neurologic dysfunction and even failed surgery [37]. Accordingly, an effective and safe barrier between the dura and scar tissue to prevent postoperative scar tissue adhesions is needed. In group B, laminar reconstruction built such a bone barrier between the dura and scar tissue that avoids laminectomy membrane formation and may prevent back pain and iatrogenic spinal stenosis. According to the results described above, ULL is a better surgical treatment for aged patients with SST/LTB than BL.

However, even if ULL has many advantages, there are still some limitations but can be solved. First, the visual field during surgery is limited by using ULL, which poses a challenge to surgeons in terms of debridement and decompression. According to our experience, TB abscesses and necrotic tissue cause compression of the spinal cord, which can be cleared using an aspirator and curettage through ULL. To remove the lesion on the non-decompression side, we tilted the operating table 30° towards the non-decompression side. Then, we could see the contralateral lesion and clear it. In addition, we tried our best to scrape away the sclerotic bone, which is regarded as a barrier for anti-TB drugs to penetrate the lesion center [38, 39]. After the removal of sclerotic bone, anti-TB drugs can seep into the focus, and TB can be cured. In a previous study, Wang, S T et al. reported that they also achieved an excellent clinical outcome without extensive anterior debridement, which also proved that extensive anterior debridement is not necessary for spinal TB [16]. Second, it was challenging to perform bone grafting though ULL. To perform a solid bone fusion, the bone block should be carefully shaped according to the size of the bone defect. We always implant a single large bone block during surgery. However, when a single bone block is challenging to implant, multiple shaped bone blocks can be selected to facilitate the fusion of the anterior interbody fusion. Third, the indications for ULL had limitations. ULL was not suitable for patients who needed a wide range of release of posterior elements for deformity correction. Hence, ULL is not recommended for patients with extensive paraspinal abscesses, multiple vertebral involvement, and rigid kyphoscoliosis. We should strictly evaluate and screen patients before surgery. However, with the development of microsurgical tools, this operation can be suitable for and cure more patients with spinal TB.

There are still some limitations in our study. First, it is a retrospective study, rather than a prospective study, the lack of randomised prospective studies meant that selection bias cannot be excluded completely. Nonetheless, the patients recruited in this study underwent a minimum 26-month follow-up. And, we hope to do a prospective observational trial to assess the risk factors associated with major complications in the ULL technique in the future study. Second, we do not clearly justify the proper indications of ULL, longer-term studies are also needed to further clarify the indications of ULL and provide more favourable clinical outcomes for elderly patients with spinal TB.

## Conclusion

In conclusion, posterior ULL debridement and bone grafting fusion combined with internal fixation is a safe and effective surgical treatment for aged patients with SST/LTB. Compared to BL, ULL is a less invasive surgical technique that leads to less surgical trauma and achieves better clinical outcomes.

## Data Availability

The datasets generated and/or analysed during the current study are not publicly available due to private information of patients but are available from the corresponding author on reasonable request.

## Abbreviations

TB: Tuberculosis
ULL: Unilateral limited laminectomy
T/TLTB: Thoracic and thoracolumbar tuberculosis
BL: Total Laminectomy
VAS: Visual Analogue Scale
ODI: Oswestry Disability Index
HREZ: Isoniazid, rifampin, ethambutol, and pyrazinamide
HRE: Isoniazid, rifampin, and ethambutol
ESR: Erythrocyte sedimentation rate
CRP: C-reaction protein
CT: Computed tomography
MRI: Magnetic resonance imaging
ASIA classification: American Spinal Injury Association classification
